# Additive value of 3T cardiovascular magnetic resonance coronary angiography for detecting coronary artery disease

**DOI:** 10.1186/s12968-018-0450-2

**Published:** 2018-04-30

**Authors:** Lijun Zhang, Xiantao Song, Li Dong, Jianan Li, Ruiyu Dou, Zhanming Fan, Jing An, Debiao Li

**Affiliations:** 1 Department of Radiology, Beijing Anzhen Hospital, Capital Medical University, Anzhenli Avenue, Chao Yang District, Beijing, 100029 China; 2 Department of Cardiology, Beijing Anzhen Hospital, Capital Medical University, Beijing, China; 3 Siemens Shenzhen Magnetic Resonance Ltd, Guangdong Shenzhen, China; 4 Biomedical Imaging Research Institute, Cedars-Sinai Medical Center, University of California, Los Angeles, USA

**Keywords:** 3 Tesla, Contrast enhanced, Coronary magnetic resonance angiography, Stress-rest perfusion imaging, Late gadolinium enhancement, Coronary artery disease

## Abstract

**Background:**

The purpose of the work was to evaluate the incremental diagnostic value of free-breathing, contrast-enhanced, whole-heart, 3 T cardiovascular magnetic resonance coronary angiography (CE-MRCA) to stress/rest myocardial perfusion imaging (MPI) and late gadolinium enhancement (LGE) imaging for detecting coronary artery disease (CAD).

**Methods:**

Fifty-one patients with suspected CAD underwent a comprehensive cardiovascular magnetic resonance (CMR) examination (CE-MRCA, MPI, and LGE). The additive diagnostic value of MRCA to MPI and LGE was evaluated using invasive x-ray coronary angiography (XA) as the standard for defining functionally significant CAD (≥ 50% stenosis in vessels > 2 mm in diameter).

**Results:**

90.2% (46/51) patients (54.0 ± 11.5 years; 71.7% men) completed CE-MRCA successfully. On per-patient basis, compared to MPI/LGE alone or MPI alone, the addition of MRCA resulted in higher sensitivity (100% vs. 76.5%, *p* < 0.01), no change in specificity (58.3% vs. 66.7%, *p* = 0.6), and higher accuracy (89.1% vs 73.9%, *p* < 0.01) for CAD detection (prevalence = 73.9%). Compared to LGE alone, the addition of CE-MRCA resulted in higher sensitivity (97.1% vs. 41.2%, *p* < 0.01), inferior specificity (83.3% vs. 91.7%, *p* = 0.02), and higher diagnostic accuracy (93.5% vs. 54.3%, *p* < 0.01).

**Conclusion:**

The inclusion of successful free-breathing, whole-heart, 3 T CE-MRCA significantly improved the sensitivity and diagnostic accuracy as compared to MPI and LGE alone for CAD detection.

## Background

Coronary artery disease (CAD) is a leading cause of mortality and morbidity around the world [1, 2]. Accurate diagnosis of CAD is important in risk stratification and in guiding clinical management [3–5]. Cardiovascular magnetic resonance (CMR) has emerged as an effective tool for the detection of CAD. CMR at both 1.5 T and 3 T has increasingly been used in clinical routine to assess myocardial ischemia and infarction caused by coronary artery stenosis using stress-rest myocardial perfusion imaging (MPI) and late gadolinium enhancement (LGE) imaging [6]. However, it is highly desirable to directly visualize coronary artery stenoses. CMR coronary angiography (MRCA) is technically challenging but has seen steady improvement in the last decade with highly promising clinical results [7–11]. At 1.5 T, several studies have shown the incremental value of MRCA when added to CMR MPI and LGE [12, 13]. The combined use of MRCA and CMR stress perfusion improved specificity for the detection of significant CAD [12]. Contrast-enhanced MRCA (CE-MRCA) at 3 T has shown excellent results in preliminary patient studies [10]. The purpose of this study was to evaluate the incremental value of CE-MRCA for the detection of CAD when added to routine CMR on 3 T.

## Methods

### Patient population

**Fig. 1:**
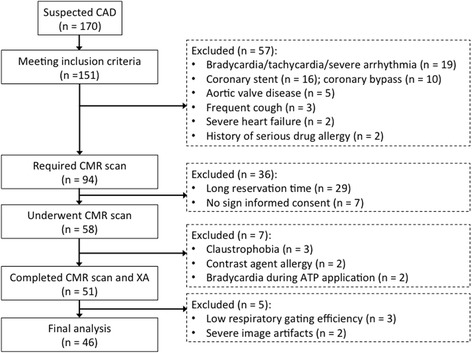
Study flow chart and reasons for exclusion of patients

From October 2015 to February 2017, consecutive patients with suspected CAD were prospectively recruited. Inclusion criteria were: (1) age 30 years or older, (2) presence of at least one major cardiovascular risk factor (smoking, diabetes, hypertension, or dyslipidemia), (3) documented stable angina. Figure 1 summarizes the study flow chart and the reasons for the exclusion of patients.

The study protocol was approved by the Ethics Committee of Beijing Anzhen Hospital. Written informed consent was obtained from the participants for publication of their individual details and images in this manuscript. The consent forms are held at the authors’ institution and are available for review by the Editor-in-Chief.

### CMR protocol

**Fig. 2:**
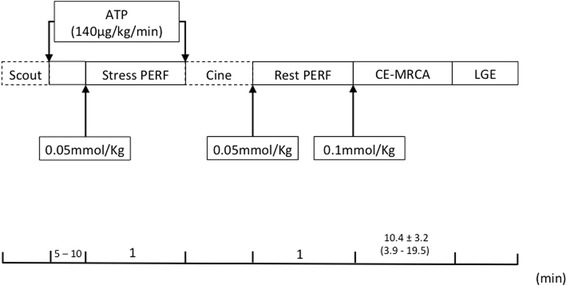
CMR Protocol

All examinations were performed on a 3 T whole-body scanner (MAGNETOM Verio, A Tim System; Siemens Healthineers, Erlangen, Germany) with a 32-element matrix coil. Detailed protocols with CE-MRCA and CMR are given in Fig. 2.

For CE-MRCA, a navigator-gated, electrocardiogram (ECG)-triggered, fat-saturated, inversion-recovery prepared segmented 3D gradient-echo sequence was employed [10]. Before examination, patients were trained to perform regular, shallow breathing and to avoid changes in depth of breathing during data acquisition [10]. Sixty seconds after the initiation of contrast agent administration (Magnevist, Bayer Healthcare,Berlin, Germany; 0.1 mmol/kg, 0.2 ml/sec), whole-heart CE-MRCA data acquisition was started. The imaging volume was prescribed in the axial plane to cover the entire heart. Prospective real-time adaptive motion correction was applied in the superior-inferior direction to compensate the respiratory motion based on the navigator signal with a correction factor of 0.6 [10]. Imaging parameters included: TR/TE = 3.0/1.4 ms, flip angle = 20°, readout bandwidth = 610 Hz/pixel, acquired voxel size = 1.3 × 1.3 × 1.3 mm^3^ and interpolated to 0.65 × 0.65 × 0.65 mm^3^. Data acquisition was accelerated by employing generalized autocalibrating partially parallel acquisitions (GRAPPA) in the phase-encoding direction with a factor of 2. A non-selective inversion pulse was applied prior to the navigator gating and data acquisition to suppress background tissues. The inversion recovery time (TI) was 200 msec.

For CMR MPI, a T1-weighted saturation-recovery fast gradient echo sequence was used with TR/TE = 165.0/1.1 ms, flip angle =12°, TI = 100 ms, FOV = 350 × 450 mm^2^, slice thickness = 8 mm. Three left ventricular (LV) short-axis slices (basal, midventricular, and apical) were acquired under maximal hyperemia achieved with 140 μg/kg/min IV adenosine infusion for 5 min, during the first pass of a bolus of 0.05 mmol/kg of contrast media (Magnevist, Bayer Healthcare) injected at 5 ml/s. During the examination, blood pressure and ECG were continuously recorded. Ten minutes after the stress perfusion, the same scan was repeated at rest.

For LGE imaging, a 2D phase-sensitive inversion recovery breath-hold sequence was used at least 10 min after the last administration of gadolinium (TR/TE = 4.1/1.56 ms, flip angle = 20°, FOV = 350 × 284 mm^2^, slice thickness = 8 mm). LGE images were acquired in two LV long-axis (two-chamber and four-chamber) views and in multiple short-axis views with a slice distance of 8 mm, covering the whole LV from base to apex.

### Imaging analysis

**Fig. 3:**
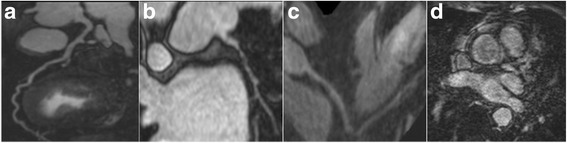
Examples of contrast enhanced magnetic resonance coronary angiography (CE-MRCA) imaging quality grades, Grade 4 = minimal artifacts, excellent signal and contrast on left anterior descending coronary artery (LAD) (**a**); Grade 3 = slight artifacts and good signal and contrast on the left circumflex coronary artery (LCX) (**b**); Grade 2 = moderate artifacts and fair signal and contrast on the right coronary artery (RCA) (**c**); Grade 1 = severe image artifacts and poor signal and contrast on LAD (**d**)

CE-MRCA was assessed by 2 experienced readers who were blinded to the patient information. Axial source images were assessed on a per-segment basis. The 15-segment American Heart Association (AHA) classification system was used for CAD diagnosis. Examples of various CE-MRCA image qualities (ImQ) are shown in Fig. 3. Images with ImQ 2 to 4 were evaluated for stenosis, and ImQ 1 was deemed non-assessable. CE-MRCA at each segment was graded on 5-point scale: 0 = normal; 1 = mild stenosis (< 50%); 2 = significant stenosis ≥50%; 3 = uninterpretable (cannot exclude significant stenosis); 4 = not visible. Segments with grades 0 and 1 were classified as negative and those with grade 2 were classified as positive. Segments with grades 3 and 4 were classified as negatives or positive depending on the results of MPI or LGE. When combining CE-MRCA and MPI or LGE, if any of the tests was positive, the overall result was deemed positive; if all were negative, the overall result was negative.

CMR perfusion images were assessed by visual comparison of stress and rest scans (17 segment American Heart Association (AHA) model, excluding the apex [14]). A perfusion deficit was determined if there were ten or more consecutive frames of apparent signal reduction. LGE images were analyzed visually using AHA segmentation, and bright segments were classified as positive.

Patients underwent x-ray coronary angiography (XA) within 1 week after CMR. The 15-segment AHA classification system was used for CAD diagnosis. Stenoses were quantitatively evaluated for segments with a diameter of > 2 mm (QuantCor software, QCA, Siemens Healthineers). Functionally significant CAD was defined as the presence of luminal stenosis ≥50%. Correspondence between LV myocardial segments and coronary arteries was determined based on the AHA model [14].

### Statistical analysis

Continuous data are reported as mean ± standard deviation. Data analysis was performed on both patient- and vessel-bases. Sensitivity and specificity were calculated using true positive (TP), true negative (TN), false positive (FP), and false negative (FN) rates using XA as reference. The McNemar test was used to calculate differences between proportions (sensitivity, specificity, and accuracy) obtained from paired observations. Receiver operating characteristic (ROC) curves were calculated to assess the diagnostic efficacy and the area under the ROC curves (AUC) were compared between different imaging methods. Cohen’s kappa statistic was used to assess inter-observer agreements. SPSS statistics (version 21, International Business Machines, Armonk, New York, USA) was used for data analysis. *P* < 0.05 was considered a significant difference.

## Results

One hundred and fifty one patients met the inclusion criteria of which 51 underwent CMR scanning. Detailed exclusion reasons were summarized in Fig. 1. The mean exam duration was 58.0 ± 7.9 min. The final analysis included 46 individuals (54.0 ± 11.5 years, 71.7% males) after excluding incomplete CE-MRCA (*n* = 5). Patient characteristics are shown in Table 1.

**Table 1:** Characteristics of the study population

	Sensitivity (%)	Specificity (%)	Accuracy (%)
Patient Basis (*N* = 46, CAD 73.9%)			
MPI	76.5 (26/34)	66.7 (8/12)	73.9 (34/46)
MPI/LGE	76.5 (26/34)	66.7 (8/12)	73.9 (34/46)
MRCA	97.1 (33/34)	91.7 (11/12)	95.7 (44/46)
CE-MRCA+MPI/LGE	100 (34/34)	58.3 (7/12)	89.1 (41/46)
LGE	41.2 (14/34)	91.7 (11/12)	54.3 (25/46)
CE-MRCA+LGE	97.1 (33/34)	83.3 (10/12)	93.5 (43/46)
Vessel Basis (*N* = 138, CAD 30.4%)			
MPI	67.9 (38/56)	82.9 (68/82)	76.8 (106/138)
MPI/LGE	67.9 (38/56)	82.9 (68/82)	76.8 (106/138)
CE-MRCA	89.3 (50/56)	89.0 (73/82)	89.1 (123/138)
CE-MRCA+MPI/LGE	94.6 (53/56)	73.2 (60/82)	81.9 113/138)
LGE	28.6 (16/56)	95.1(78/82)	68.1 (94/138)
CE-MRCA+LGE	89.3 (50/56)	84.1(69/82)	86.2 (119/138)

### Image quality and assessability of MRCA

A total of 90.2% (46/51) of CE-MRCA studies were included in data analysis. Five patients were excluded: CE-MRCA was aborted in three patients due to extremely low respiratory gating efficiency (navigator efficiency< 20% by the time half of the imaging data were collected); severe motion artifacts were present in two patients as evaluated by an experienced radiologist. The mean scan time was 10.4 ± 3.2 min, ranging from 3.9 to 19.5 min. Patients did not receive medicine to reduce their heart rate (HR), thus 22 patients had a HR > 65 bpm and 24 patients had HR ≤ 65 bpm, with a mean HR of 67.3 ± 9.6 bpm. The average navigator respiratory gating efficiency was 35.0 ± 9.1%. CE-MRCA was acquired during diastole in 21 patients (77% with HR ≤ 65 bpm) and during systole in 25 (67% with HR > 65 bpm). The average scan time of patients with diastolic acquisition was similar to that of systolic acquisition (10.3 ± 2.6 min vs. 10.4 ± 3.8 min, *p* = 0.28).

Significant stenosis was found in 73.9% patients (34/46) based on MRCA. The kappa value for inter-observer agreement on per-patient basis for the identification of significant stenosis was 0.78 (95% CI, 0.55–1.0). Of the 34 patients, 16 (47.1%) had 1-vessel CAD, 11 (32.4%) had 2-vessel CAD, and 7 (20.6%) had 3-vessel CAD. On per-vessel basis, 42.8% (59/138) vessels had significant stenosis detected by MRCA, including 30.5% right coronary artery (RCA) (18/59), 40.7% left anterior descending artery (LAD) (24/59), and 28.8% left circumflex artery (LCX) (17/59).

Of the total 690 segments, 529 segments had diameter > 2 mm and 96.4% (510/529) were judged to have adequate image quality (ImQ 4 = 391, ImQ 3 = 101, ImQ 2 = 18) with an average ImQ of 3.7 ± 0.5. For stenosis grade, of 510 segments, 410 (80.4%) had grade 0 or 1, 89 (17.5%) had grade 2. Eleven segments had grades 3 (uninterpretable) or 4 (invisible).

On per-patient basis, CE-MRCA had a sensitivity of 97.1%, specificity of 91.7%, accuracy of 95.7%, and AUC of 0.94 (95% CI, 0.83–0.99). On per-vessel basis, CE-MRCA had a sensitivity of 89.3%, specificity of 89.0%, accuracy of 89.1%, and AUC of 0.89 (95% CI, 0.83–0.94).

### Combination of CE-MRCA with MPI

The mean heart rates were 91 ± 11.4 bpm (ranging from 70 to 120 bpm) during stress perfusion and 67 ± 9.5 bpm (ranging from 50 to 86 bpm) during rest perfusion. The average increased HR was 23 ± 8.5 bpm (ranging from 9 to 42 bpm). On per-patient basis, the kappa value for inter-observer agreement for the identification of perfusion defect was 0.73 (95% CI, 0.52–0.93).

Of the total 736 segments in the 46 patients, 23.2% (171/736) was positive on MPI alone, corresponding to 52 main branches (RCA 21, LAD 16, and LCX 15). After adding CE-MRCA, the positive results increased to 75 main branches (RCA 25, LAD 29, and LCX 21).

**Fig. 4:**
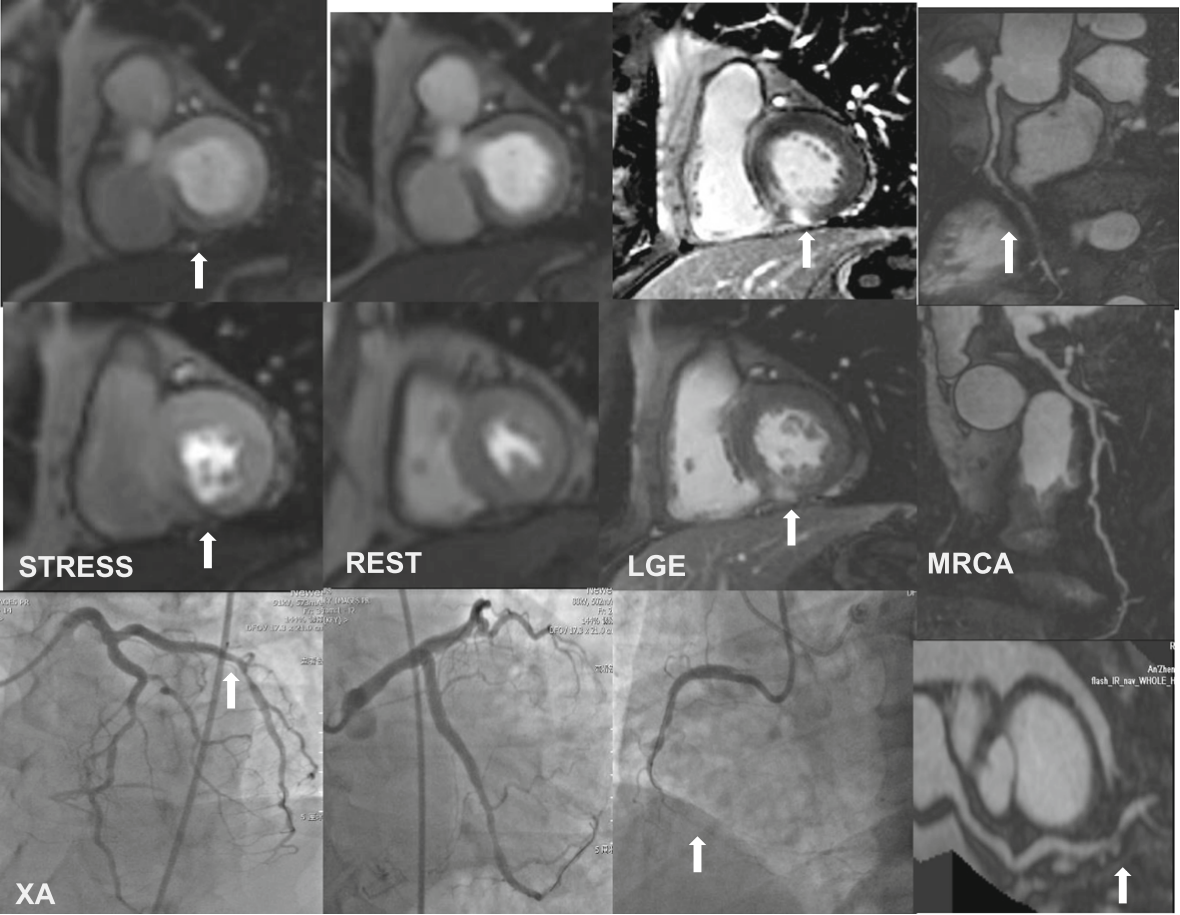
One case illustrating myocardial perfusion imaging (MPI), late gadolinium enhancement (LGE), CE-MRCA and x-ray angiography (XA) angiographic findings in patient with coronary artery disease (CAD), Male/60 yrs. MPI (+) + MRCA (+) = combination (+). LGE (+) + MRCA (+) = combination (+). MPI/LGE (+) + MRCA (+) = combination (+). Corresponding XA shows significant RCA and LCX stenoses

**Fig. 5:**
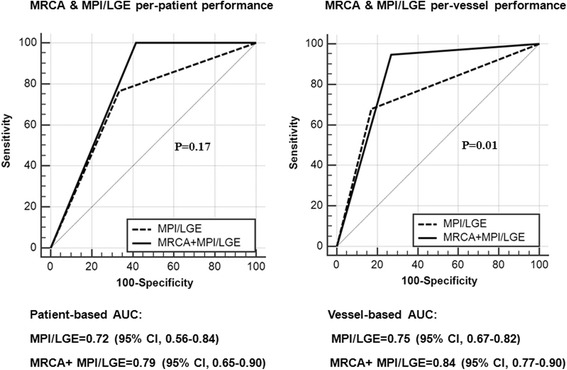
Receiver operator curve (ROC) curve analyses for MPI/LGE alone and combination of CE-MRCA with MPI/LGE on per-patient and per-vessel bases

The diagnostic performance of combined CE-MRCA and MPI compared to MPI alone was assessed in 46 patients and 138 vessels respectively. Results are given in Table 2. Compared to MPI alone, adding CE-MRCA to MPI showed higher sensitivity (100% vs. 76.5%, *p* < 0.01), similar specificity (58.3% vs. 66.7%, *p* = 0.6), and higher accuracy (89.1% vs. 73.9%, p < 0.01) on per-patient basis (Fig. 4). ROC analysis showed that there was no significant difference between the combination of CE-MRCA and MPI (AUC =0.79; 95% CI, 0.65–0.90), compared with MPI alone (AUC = 0.72; 95% CI, 0.56–0.84) (*p* = 0.17). AUC was improved in vessel-based analysis (0.84 [95% CI, 0.77–0.90] vs. 0.75 [95% CI, 0.67–0.82], *p* = 0.01) (Fig. 5).

**Table 2:** Diagnostic accuracy of the different imaging methods and their combination on per-patient and per-vessel bases

Number of patients (n)	46
Male sex	33 (71.7%)
Age (yrs)	54 ± 11.5
Body-mass index (kg/m^2^)	26 ± 3.2
Hypercholesterolemia	13 (28.2%)
Hypertension	29 (63.0%)
Diabetes mellitus	8 (17.4%)
Positive smoking history	17 (37.0%)
Current smoker	16 (34.8%)
Family history of CAD	6 (13.0%)

*CAD* coronary artery disease, *MPI* stress-rest myocardial perfusion imaging, *LGE* late gadolinium enhancement, *CE-MRCA* contrast enhanced magnetic resonance coronary angiography

**Fig. 6:**
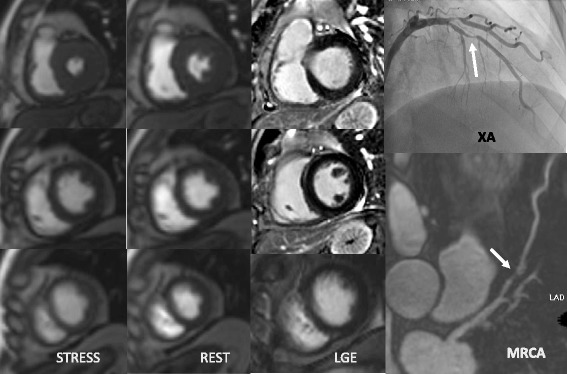
One case illustrating MPI, LGE, CE-MRCA and XA angiographic findings in patient with CAD. Male/42 yrs. MPI (−) + CE-MRCA (+) = combination (+). LGE (−) + CE-MRCA (+) = combination (+). MPI/LGE (−) + CE-MRCA (+) = combination (+). Corresponding XA shows significant LAD stenosis, correctly identified by CE-MRCA.

In MPI alone, 4 patients were FPs and 8 patients were FNs. By adding CE-MRCA, the number of FPs was increased by 1 and number of FNs was decreased from 8 to 0 (Fig. 6).

### Combination of MRCA with LGE

Based on LGE alone, 15 patients were founded positive and 31 patients were negative. Adding CE-MRCA to LGE, 35 patients were founded positive and 11 patients were negative. Although the number of FPs was slightly increased (from 1 to 2), the number of FNs was dramatically decreased from 20 to 1, compared to LGE alone (Fig. 6).

**Fig. 7:**
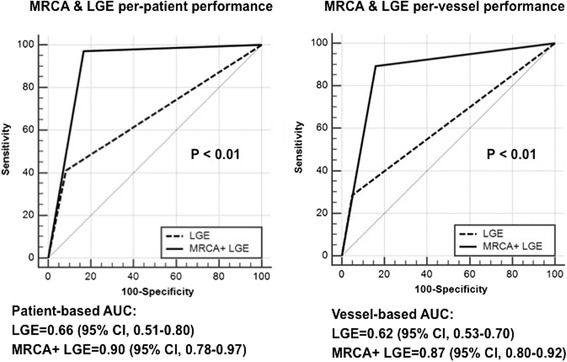
ROC curve analysis for LGE alone and combination of CE-MRCA with LGE on per-patient and per-vessel bases

Adding CE-MRCA to LGE showed significantly higher sensitivity (97.1% vs. 41.2%, *p* < 0.01) translating into better diagnostic performance (AUC = 0.90 [95% CI, 0.78–0.97] vs. 0.66 [95% CI, 0.51–0.80], *p* < 0.01) (Fig. 7). Although specificity was inferior (83.3% vs. 91.7%, *p* = 0.02), diagnostic accuracy was significantly increased from 54.3% to 93.5%, compared to LGE alone (Fig. 4). The same trends were also found in vessel-based analysis (Table 2).

### Combination of CE-MRCA with MPI/LGE

In the 46 patients, 23.2% (171/736) was positive on MPI/LGE. The diagnostic performance for the combination of CE-MRCA and MPI/LGE compared to MPI/LGE are given in Table 2. Compared to MPI/LGE alone, adding CE-MRCA showed higher sensitivity (100% vs. 76.5%, *p* < 0.01) and similar specificity (58.3% vs. 66.7%, *p* = 0.6). Overall accuracy was improved from 73.9% to 89.1% on per-patient basis (Fig. 4). ROC analysis showed that there was no significant difference between the combined CE-MRCA and MPI/LGE (AUC = 0.79; 95% CI, 0.65–0.90), compared with MPI/LGE (AUC = 0.72; 95% CI, 0.56–0.84) (*p* = 0.17). AUC was improved on per-vessel basis (0.84 [95% CI, 0.77–0.90] vs. 0.75 [95% CI, 0.67–0.82], *p* = 0.01) (Fig. 5).

It should be pointed out that these results were the same as that of MPI/LGE compared to MPI alone. A positive diagnosis is obtained when either of MPI or LGE is positive. Positive LGE in comparison to negative MPI may result from small subendocardial infarcts. Apparently it was not the case in this patient population.

## Discussion

To our knowledge, this is the first study to evaluate the additive value of 3D whole-heart CE MRCA to conventional CMR MPI and LGE for detecting CAD on a 3 T CMR system. We demonstrated that the integration of CE-MRCA into a comprehensive stress-rest MPI and LGE protocol significantly improved sensitivity and diagnostic accuracy. These results conflict with the previous study at 1.5 T [15], in which MRCA did not increase the overall diagnostic accuracy. Possible reasons include the enhanced contrast between blood and background and increased image quality due to contrast enhanced imaging and shorter scan time on 3 T as compared to non-contrast-enhanced imaging at 1.5 T. CE-MRCA increased the number of assessable coronary artery segments in comparison to unenhanced MRCA [16], especially distal segments [17]. Sommer et al. [18] and Bi et al. [19] demonstrated a significant increase in SNR from 1.5 to 3.0 T. Liu et al. [20] and Prompona et al. [21] found significant increase in contrast-to-noise ratio at 3.0 T as compared to 1.5 T. Indeed, 19% of MRCA at 1.5 T had poor image quality [15] as compared to 3.6% in this study at 3 T.

Another difference between this study and the previous studies at 1.5 T is the definition of significant stenosis. In this study, significant stenosis was defined as luminal narrowing of ≥50% as determined on XA, while in previous studies, significant stenosis was defined as luminal narrowing of ≥90% stenosis or fractional flow reserve (FFR) ≤ 0.80 by Bettencourt N et al. [15]; or luminal narrowing of ≥70% by Ripley DR et al. [22].

In this study, adding CE-MRCA to MPI and LGE reduced the incidents of false negative (from 8 to 0). These 8 false negative patients were confirmed by coronary angiography as having 50–70% stenosis (*n* = 4), 70–80% stenosis (*n* = 2), chronic total occlusion with good collateral circulation (*n* = 2). CE-MRCA accurately identified these stenoses without myocardial ischemia or infarction, thereby reducing false negatives and improving the sensitivity of diagnosis. By adding CE-MRCA to LGE, 19 of the 20 false negatives were correctly depicted by CE-MRCA.

In this study, there were 4 false-positives using MPI. Among the four patients, three had microvascular coronary dysfunction patients, showing extensive annular ischemia in the endocardium but no coronary artery stenosis on XA.

Therefore, although the detection of the morphologically significant coronary stenosis and that of hemodynamically significant coronary stenosis are clinically different, but this study shows that CE-MRCA can detect the morphologically significant coronary stenosis in patients without hemodynamic significant stenosis, so as to avoid the omission of severe stenosis, so that patients can get the corresponding clinical treatment as early as possible; and CE-MRCA can screened out the patients without three main coronary artery morphologically stenosis but with hemodynamic stenosis, so that these patients can avoid invasive examination.

### Study limitations

In this study, 10% patients excluded due to CE-MRCA imaging issues, 3/51 patients were not able to complete CE-MRCA due to low respiratory gating efficiency and 2/51 patients had severe motion artifacts. They were not included in data analysis. 26% of CE-MRCA segments were not analysable for small diameter (161/690) and severe image artifacts and poor signal and contrast (19/690). This is a single center study with very small patient numbers. A prospective, multicenter study with a larger sample size is needed to further confirm the findings of the study.

## Conclusion

On 3 T, integration of successful 3D whole-heart CE-MRCA into a comprehensive stress-rest myocardial perfusion or LGE protocol significantly improved sensitivity and diagnostic accuracy for detection of CAD.

## Abbreviations

AHA: American Heart Association
AUC: The area under the ROC curves
CAD: Coronary artery disease
CE-MRCA: Contrast-enhanced magnetic resonance coronary angiography
CMR: Cardiovascular magnetic resonance
ECG: Electrocardiogram
FFR: Fractional flow reserve
FN: False negative
FP: False positive
HR: Heart rate
ImQ: Image quality
LAD: Left anterior descending coronary artery
LCX: Left circumflex coronary artery
LGE: Late gadolinium enhancement
LV: Left ventricle/left ventricular
MPI: Stress/rest myocardial perfusion imaging
RCA: Right coronary artery.
ROC: Receiver operating characteristic
TI: Inversion time
TN: True negative
TP: True positive
XA: X-ray coronary angiography
